# Mitochondrial Distribution Patterns and Remodeling During *In Vitro* Maturation of Alpaca (*Vicugna pacos*) Oocytes

**DOI:** 10.3390/biology15141206

**Published:** 2026-07-21

**Authors:** Paula Quijano, Alejandra Ugarelli, José Sánchez, Omar Ramirez, Alexei Santiani, Shirley Evangelista-Vargas

**Affiliations:** 1 Grupo de Investigación en Biología y Biotecnología Reproductiva Animal, Universidad Científica del Sur, Lima 15067, Peru; pquijanos@cientifica.edu.pe (P.Q.); mugarelliga@cientifica.edu.pe (A.U.); 100032658@cientifica.edu.pe (J.S.); 100035129@cientifica.edu.pe (O.R.); 2 Laboratory of Animal Reproduction, Faculty of Veterinary Medicine, Universidad Nacional Mayor de San Marcos, Lima 15021, Peru; asantiania@unmsm.edu.pe

**Keywords:** alpaca oocytes, mitochondrial activity, mitochondrial distribution, *in vitro* maturation, cytoplasmic maturation, South American camelids

## Abstract

Mitochondrial distribution and activity are closely associated with cytoplasmic maturation and developmental competence in mammalian oocytes; however, limited information is available regarding these processes in *Vicugna pacos*. This study aimed to characterize mitochondrial activity, aggregation, and distribution patterns in immature and *in vitro* mature *Vicugna pacos* oocytes. Mitochondrial fluorescence intensity ranged from 21.2 to 126 units, with mean values decreasing from grade I to IV. No significant differences were observed between immature (66.8 ± 22.7) and mature (59.2 ± 23.5) oocytes. Three distinct distribution patterns were identified: homogeneous non-peripheral (HoNP), heterogeneous peripheral (HeP), and heterogeneous non-peripheral (HeNP), with HeNP oocytes exhibiting the highest activity. During maturation, fluorescence intensity increased in HeNP and HeP but decreased in HoNP, suggesting distinct mitochondrial responses. Aggregation shifted from smooth in immature oocytes to granular in mature ones, and these two traits—HeNP distribution and granular aggregation—were consistently associated with higher mitochondrial activity. Overall, 78% of oocytes showed aggregation. These results demonstrate that mitochondrial remodeling accompanies oocyte maturation and that aggregation and distribution patterns may serve as biomarkers of oocyte quality. Further studies linking these features with fertilization and embryo development are needed to validate their predictive role in assisted reproduction of alpacas.

## Simple Summary

Improving assisted reproductive technologies in alpacas requires reliable indicators of oocyte quality. In this study, we evaluated mitochondrial behavior—organelles that generate energy—during oocyte maturation. We analyzed their activity, spatial distribution, and aggregation patterns before and after *in vitro* maturation. Configurations such as central clustering (HeNP distribution) and granular aggregation were associated with higher activity levels. These traits reflect cytoplasmic maturity and could serve as markers for selecting oocytes with greater developmental potential. This knowledge may support embryo production techniques and genetic conservation programs in South American camelids.

## 1. Introduction

Improving reproductive biotechnologies in alpacas (*Vicugna pacos*) requires a deeper understanding of gamete physiology and reliable biomarkers of developmental competence. While sperm quality has been extensively assessed using CASA and flow cytometry in camelids, including alpacas [1,2], research on oocyte quality in this species remains limited. At present, oocyte selection relies mainly on morphological criteria, such as cumulus cell layers and cytoplasmic appearance [3]. Although the inherently low oocyte recovery rates in alpaca *in vitro* production (IVP) systems sometimes necessitate culturing all recovered oocytes, prioritizing those with optimal morphology (Grades I and II) remains a standard and highly recommended practice to maximize overall efficiency. However, because these visual features often fail to accurately reflect the true functional and developmental competence of the oocyte, there is a critical need to complement current selection strategies with subcellular biomarkers. By addressing this limitation, our study investigates mitochondrial activity as a potential tool to refine oocyte selection.

Reproductive efficiency in South American camelids is notably low: approximately 50% of females do not produce offspring within a year, and up to 50% of embryos are lost during the first month of gestation, specifically encompassing the critical pre-implantation and early implantation stages. Furthermore, only ~35% of females become pregnant after mating [4]. *In vivo* embryo production via superovulation yields an average of ~4.75 embryos per female, and early attempts at *in vitro* embryo production in alpacas resulted in only ~8% transferable embryos. More recent studies have reported blastocyst rates of ~20% [5]. These statistics highlight the urgent need for improved oocyte selection and optimized culture protocols.

Recent evidence emphasizes the relevance of subcellular markers such as mitochondrial activity, distribution, and morphology in determining oocyte quality. Mitochondria are essential for ATP production, calcium regulation, and redox balance—key processes that sustain meiosis resumption, fertilization, and early embryogenesis [6,7,8]. Consequently, mitochondrial parameters have been proposed as more reliable indicators of cytoplasmic maturation and developmental competence than morphology alone.

In addition, mitochondrial quality control mechanisms—including fission/fusion dynamics, biogenesis, and mitophagy—play a pivotal role in oocyte maturation and reproductive efficiency, as demonstrated in bovine models [9]. Conversely, altered mitochondrial distribution and function under stress conditions, such as heat stress, are associated with reduced oocyte competence in mammals [9].

Mitochondrial distribution patterns are of particular interest. In several mammalian species, mitochondria localize to regions of high metabolic demand or accumulate peripherally, often in association with gap junction communication with cumulus cells [10]. Studies in canine, bovine, porcine, and human oocytes have described distinct distribution patterns—including homogeneous, heterogeneous, peripheral, central, smooth, and granular—that are linked to oocyte quality and developmental potential [11,12,13,14].

In South American camelids, despite advances in *in vitro* maturation (IVM) and fertilization (IVF) protocols [15]—including ICSI and various follicular aspiration techniques—the detailed dynamics of mitochondria behavior in alpaca oocytes remain largely unexplored. Oocyte recovery by ovum pick-up (OPU) in llamas has achieved blastocyst rates of approximately 35%—one of the highest reported for South American camelids. While these results are linked to the completion of *in vivo* maturation, comprehensive insights into cytoplasmic maturation are still missing [16].

Therefore, the objective of this study was to evaluate mitochondrial activity, aggregation, and spatial distribution in immature and *in vitro* matured alpaca oocytes. These findings aim to address a critical knowledge gap, support the development of evidence-based IVM systems, and ultimately enhance oocyte selection strategies and embryo production outcomes in alpaca reproductive biotechnologies.

## 2. Materials and Methods

### 2.1. Collection of Cumulus–Oocyte Complexes (COCs) and Oocyte Handling

Alpaca ovaries were obtained from a slaughterhouse in Huancavelica, Peru. Immediately after slaughter, they were excised, rinsed with physiological saline, and placed in sterile bags containing 0.9% NaCl. Samples were transported at 4 °C to Lima within 10–16 h [17] and processed at the laboratory of Universidad Científica del Sur. In the laboratory, ovaries were washed with phosphate-buffered saline (PBS), and excess connective tissue and visible blood vessels were removed. Follicles measuring 3–4 mm in diameter were aspirated using a sterile 5 mL syringe fitted with a 21G 1½” needle. The follicular fluid was collected in 15 mL Falcon tubes and allowed to sediment at 37 °C. The resulting pellet was recovered using a Pasteur pipette and transferred to 60 × 15 mm Petri dishes maintained at 37 °C. COCs were subsequently washed in 100 × 20 mm Petri dishes by adding 3–4 drops (300–400 µL) of prewarmed small ruminant wash medium (Stroebech Media™, Aalestrup, Denmark) at 38.8 °C. COCs quality was assessed under a stereomicroscope (40×).

**Figure 1:**
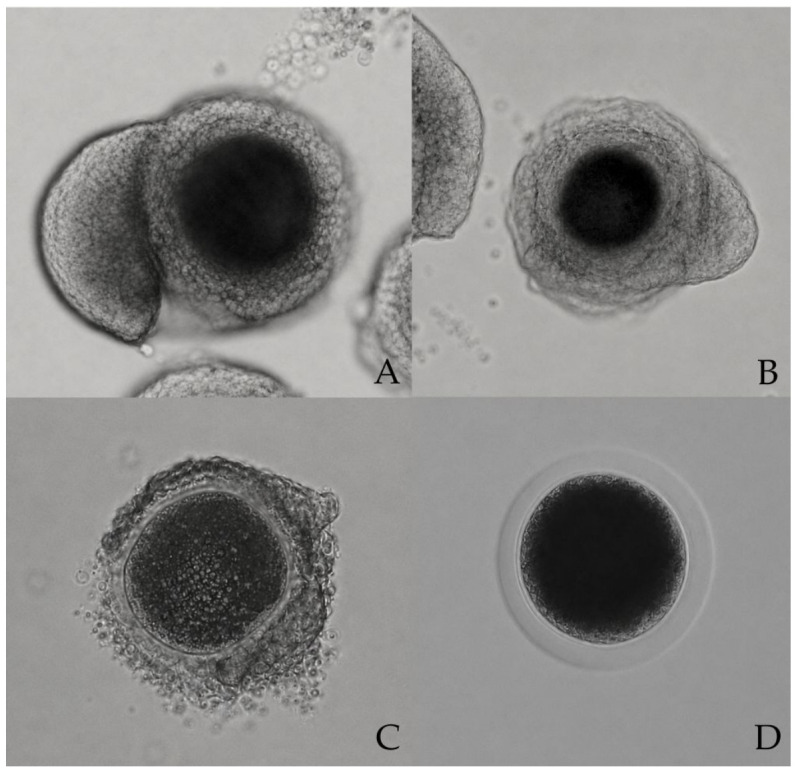
Representative images of immature alpaca oocytes classified by quality: (**A**) Grade I; (**B**) Grade II; (**C**) Grade III; (**D**) Grade IV.

COCs were graded into four quality categories according to cumulus cell layers and cytoplasmic appearance, following criteria described for alpaca [18]. Grade I: ≥5 compact cumulus cell layers, homogeneous cytoplasm. Grade II: 2–4 layers, homogeneous cytoplasm. Grade III: ≤1 layer or partial denudation, with cytoplasmic vacuolization. Grade IV: completely denuded or with granular/irregular cytoplasm (Figure 1).

To establish a robust baseline of initial quality, a total of 120 immature oocytes (30 per quality grade) were selected for immediate mitochondrial assessment. For these immature oocytes, cumulus cells were mechanically removed without the use of enzymatic agents, and the oocytes were washed twice in PBS before staining. In parallel, to evaluate maturation competence, an additional 136 immature oocytes were classified prior to IVM and cultured in separate groups according to their initial grade (34 oocytes per grade). Groups of 17 oocytes were incubated in 500 μL of small ruminant *in vitro* maturation medium (Stroebech Media™, Aalestrup, Denmark) at 38.5 °C, 6% CO_2_ and high humidity for 36 h, following the manufacturer’s instructions. After incubation, denuded oocytes were examined for the presence of a polar body as an indicator of nuclear maturation. Maturation outcomes were 22/34 (64.7%) for Grade I, 21/34 (61.8%) for Grade II, 13/34 (38.2%) for Grade III, and 10/34 (29.4%) for Grade IV. To prevent statistical bias from unequal group sizes during comparison, a randomly selected subset of exactly 10 mature oocytes per grade was then utilized for mitochondrial assessment.

### 2.2. Evaluation of Mitochondrial Activity, Aggregation, and Distribution

Mitochondrial activity was assessed via live staining using MitoTracker^®^ Red CMXRos (Molecular Probes, Eugene, OR, USA) at a final concentration of 100 nM [19]. To ensure the accurate capture of active mitochondrial membrane potential, exclusively live oocytes were evaluated. Immature oocytes were stained and assessed immediately following mechanical denudation post-recovery, whereas mature oocytes were stained and evaluated immediately upon completion of the 36-h *in vitro* maturation (IVM) period and their subsequent denudation. Briefly, 99.5 µL PBS was mixed with 0.5 µL of 20 µM stock solution, and four oocytes were mounted per slide. Coverslips were positioned using silicone spacers to prevent compression. Preparations were incubated in dark at 28 °C for 20 min.

This mounting method, adapted from feline protocols [19], incorporates silicone supports to protect fragile alpaca oocytes, thereby maintaining their three-dimensional integrity during imaging. Slides were analyzed using an inverted fluorescence microscope (EVOS M5000, Thermo Fisher Scientific, Bothell, WA, USA) equipped with a Texas Red 2.0 Light Cube (Thermo Fisher Scientific, Bothell, WA, USA) (excitation: 585/29 nm; emission: 628/32 nm). Digital images were captured and fluorescence intensity (pixels) quantified with ImageJ software (version 1.54g, National Institutes of Health, Bethesda, MD, USA). To account for inherent ooplasmic autofluorescence, unstained control oocytes were evaluated under identical microscope settings prior to the analysis. The baseline fluorescence intensity obtained from these negative controls was utilized for background normalization during the quantitative image analysis in ImageJ, ensuring that the recorded signals accurately reflected the specific binding of the fluorophore.

Mitochondrial distribution patterns were classified as: homogeneous non-peripheral (uniform distribution throughout the cytoplasm), heterogeneous peripheral (irregular clusters predominantly near the cortex), and heterogeneous non-peripheral (clusters mainly in the central cytoplasmic). Mitochondrial aggregation patterns were defined as: granular (small, punctate clusters), and smooth (diffuse, evenly spread distribution). It is important to emphasize the methodological distinction between these two parameters: ‘distribution’ evaluates the global spatial localization of mitochondria across the entire ooplasm, whereas ‘aggregation’ specifically refers to the localized structural clustering and micro-organization of these organelles, functioning as an independent but complementary variable. These criteria adapted descriptions from canine and bovine oocytes [11,12], to alpacas, where no previous categorization had been reported. Image scoring was based on visual assessment and independently evaluated by two observers. Oocytes exhibiting more than one mitochondrial distribution feature were classified according to the predominant fluorescence pattern observed throughout the ooplasm.

### 2.3. Data Analysis

Descriptive statistics were used to summarize mitochondrial activity, distribution, and aggregation. Fluorescence intensity values were expressed as mean ± standard deviation (SD) and visualized with boxplots. Data distribution was assessed by the Shapiro–Wilk test. One-way ANOVA followed by Tukey’s post hoc test was used to compare fluorescence intensity across COCs grades (I–IV). Independent-samples *t*-test compared immature vs. mature oocytes. Associations between categorical variables (distribution vs. COCs grade; aggregation vs. COCs grade; activity vs. maturation stage) were analyzed using chi-square tests. Statistical analyses were conducted in SPSS v.25 (IBM Corp., Armonk, NY, USA), with significance set at *p* < 0.05.

## 3. Results

**Figure 2:**
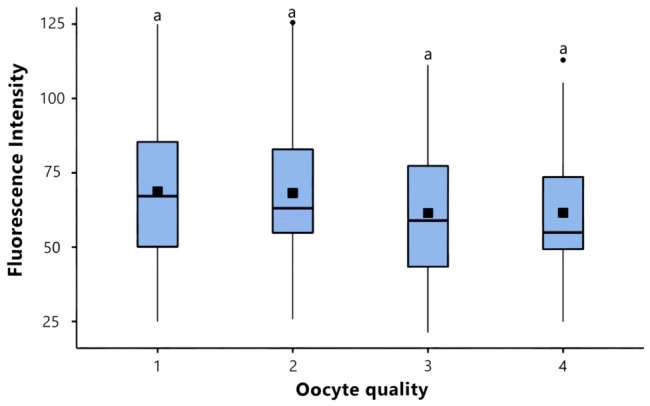
Boxplot of mitochondrial fluorescence intensity (arbitrary units, pixels) in alpaca oocytes classified by morphological quality grade (I–IV). Each box represents the interquartile range, the horizontal line indicates the median, and whiskers show the minimum and maximum values. Data are expressed as mean ± SD. Boxes with identical letters do not differ significantly (*p* > 0.05).

**Figure 3:**
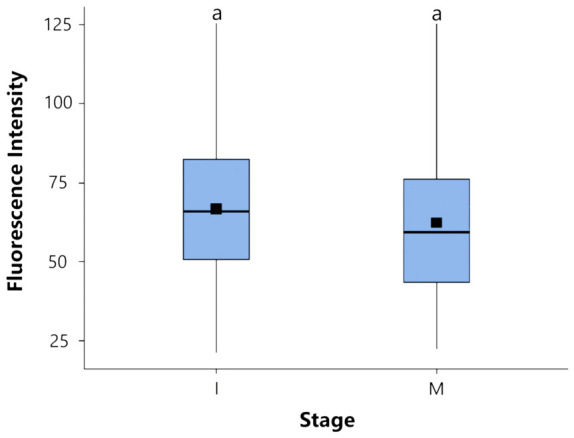
Boxplot of mitochondrial fluorescence intensity (arbitrary units, pixels) in alpaca oocytes according to maturation stage. “I” = immature oocytes (*n* = 120); “M” = *in vitro* matured oocytes (*n* = 40). Each box represents the interquartile range, the horizontal line indicates the median, and whiskers show minimum and maximum values. Data are expressed as mean ± SD. Boxes with identical letters do not differ significantly (*p* > 0.05).

The fluorescence intensity associated with mitochondrial activity ranged from 21.2 to 126 pixels across all oocytes evaluated. For descriptive purposes, values are reported as mean ± SD; however, the boxplots shown in Figure 2 and Figure 3, display medians with interquartile ranges. Mean values by quality grade were 68.7 ± 22.3 pixels for Grade I, 68.1 ± 24.9 for Grade II, 61.4 ± 23.8 for Grade III, and 61.6 ± 20.1 for Grade IV (Figure 2).

When maturation stage was considered, immature oocytes showed a mean fluorescence of 66.8 ± 22.7 pixels, while mature oocytes averaged 59.2 ± 23.5 pixels. This difference was not statistically significant (Figure 3).

**Figure 4:**
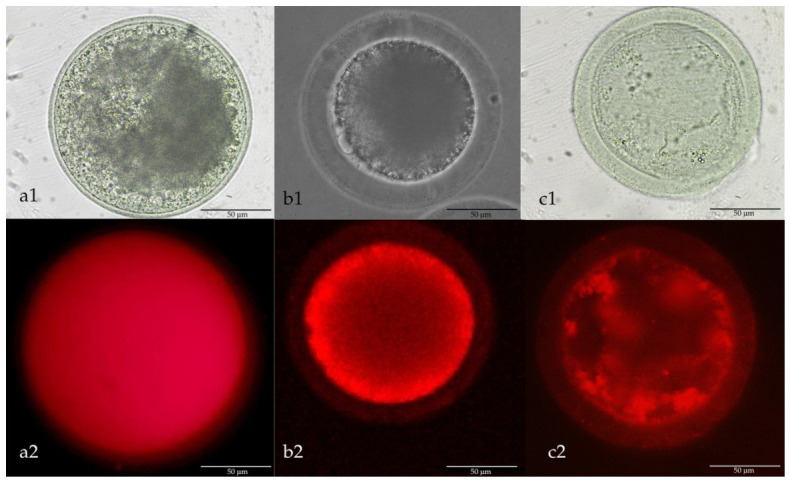
Representative bright-field and fluorescence micrographs illustrating mitochondrial distribution patterns in alpaca oocytes. Oocytes exhibiting homogeneous non-peripheral (HoNP) mitochondrial distribution pattern are shown under bright-field microscopy (**a1**) and in the corresponding fluorescence image (**a2**). Oocytes exhibiting a heterogeneous peripheral (HeP) mitochondrial distribution pattern are shown under bright-field microscopy (**b1**) and in the corresponding fluorescence image (**b2**). Oocytes exhibiting a heterogeneous non-peripheral (HeNP) mitochondrial distribution pattern are shown under bright-field microscopy (**c1**) and in the corresponding fluorescence image (**c2**). Images “a” and “c” correspond to immature oocytes, whereas image “b” depicts an *in vitro* matured oocyte.

**Figure 5:**
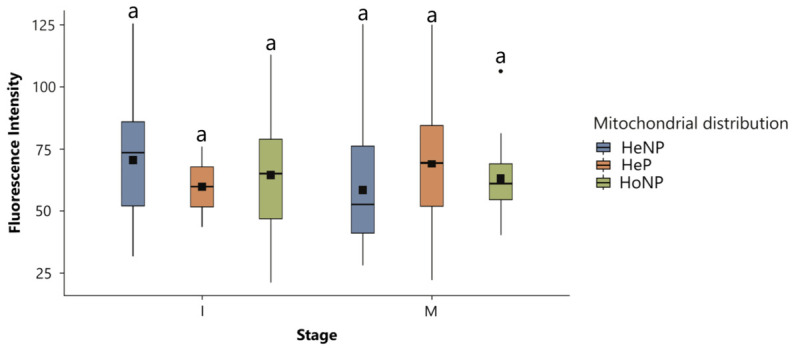
Boxplot of mitochondrial fluorescence intensity (arbitrary units, pixels) according to distribution pattern and maturation stage in alpaca oocytes. Each box represents the interquartile range, the horizontal line indicates the median, and whiskers show the minimum and maximum values. Data are expressed as mean ± SD. Boxes with identical letters do not differ significantly (*p* > 0.05).

Three mitochondrial distribution patterns were identified in both immature and mature oocytes: homogeneous non-peripheral (HoNP), heterogeneous peripheral (HeP), and heterogeneous non-peripheral (HeNP) (Figure 4). In immature oocytes, HeNP displayed higher fluorescence intensity than both HeP and HoNP. In mature oocytes, fluorescence intensity increased in HeNP and HeP but decreased in HoNP (Figure 5).

**Figure 6:**
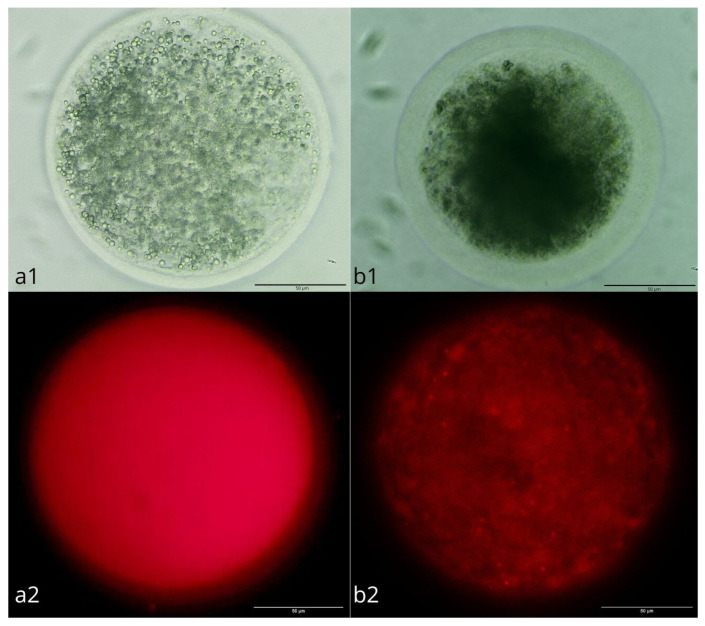
Representative bright-field and fluorescence micrographs showing mitochondrial aggregation patterns in alpaca oocytes. (**a1**,**a2**) Smooth mitochondrial distribution pattern observed under bright-field (**a1**) and fluorescence microscopy (**a2**). (**b1**,**b2**) Granular mitochondrial distribution pattern observed under bright-field (**b1**) and fluorescence microscopy (**b2**).

**Figure 7:**
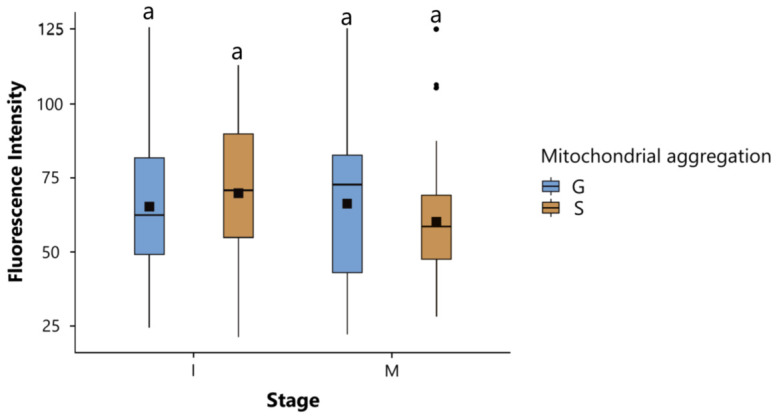
Boxplot of mitochondrial fluorescence intensity (arbitrary units, pixels) in alpaca oocytes according to aggregation pattern (smooth vs. granular) and maturation stage (immature vs. mature). Each box represents the interquartile range, the horizontal line indicates the median, and whiskers show the minimum and maximum values. Data are expressed as mean ± SD. Boxes with identical letters do not differ significantly (*p* > 0.05).

Mitochondrial aggregation was observed in 78% of oocytes and classified as either smooth or granular (Figure 6). In immature oocytes, the smooth pattern showed slightly higher fluorescence intensity than the granular pattern. In contrast, in mature oocytes the granular pattern showed higher fluorescence intensity (Figure 7).

## 4. Discussion

The present study evaluated mitochondrial activity, distribution, and aggregation in immature and *in vitro* matured alpaca oocytes, providing novel insights into cytoplasmic maturation processes in this species. Interestingly, fluorescence intensity did not differ significantly between immature and mature oocytes, a finding that contrasts with observations in other species such as dogs [11], where mitochondrial activity increases following *in vitro* maturation (IVM). Mitochondrial activity typically rises in response to heightened energy demands during oocyte maturation, particularly during meiotic resumption and extrusion of the first polar body [20]. In ruminants, mature oocytes often harbor greater amounts of mtDNA compared to their immature counterparts, suggesting an increase in mitochondrial content [21]. Similarly, equine oocytes exhibit enhanced mitochondrial activity prior to ovulation [8]. Conversely, studies in human oocytes have reported minimal changes in mitochondrial activity following maturation [22], which is consistent with the plateau observed in alpacas. One possible explanation is that oocytes obtained post-mortem may already present compromised metabolic competence, masking the expected rise in mitochondrial activity [23]. Additionally, while *in vitro* handling such as the 20-min incubation at 28 °C could induce minor metabolic shifts, this protocol was strictly and uniformly applied to all experimental groups following established methodologies for fragile oocytes, ensuring comparative validity. Furthermore, it is critical to emphasize that the assessment of mitochondrial activity was performed using MitoTracker Red CMXRos on exclusively live oocytes [19]. Unlike purely structural probes, the intracellular accumulation of this specific fluorophore is inherently dependent upon active mitochondrial membrane potential. Consequently, the quantified fluorescence intensity in our study accurately reflects the functional, metabolically active state of the mitochondria rather than their mere physical presence, validating its correlation with the observed spatial distribution patterns.

Our results revealed species-specific patterns of mitochondrial distribution in alpaca oocytes, which we categorized as homogeneous non-peripheral (HoNP), heterogeneous peripheral (HeP), and heterogeneous non-peripheral (HeNP). This classification differs from that described in canine oocytes, where mitochondrial distributions during IVM are generally classified as either smooth or granular homogeneous [10]. Despite these taxonomic differences, the resemblance in heterogeneity patterns between alpaca and canine oocytes suggests that comparable mitochondrial remodeling processes may occur under *in vitro* conditions [23]. The HeNP pattern was particularly associated with elevated mitochondrial activity, especially in immature oocytes, suggesting a greater level of metabolic readiness. Similar central aggregations of mitochondria have been documented in other species and are often linked to cytoplasmic regions with high biosynthetic demand [24]. The increased mitochondrial activity observed during maturation in HeNP and HeP groups aligns with reports from bovine and ovine oocytes, where IVM induces mitochondrial redistribution toward metabolically active regions [21]. In contrast, the reduced activity observed in HoNP oocytes may reflect limited cytoplasmic competence and, potentially, lower developmental potential [25].

Interestingly, alpaca oocytes did not exhibit a consistent transition in mitochondrial distribution from immature to mature stages. This contrasts with findings in domestic cats, where mitochondrial localization shifts from peripheral to more homogeneous patterns during maturation [26]. These differences may reflect fundamental distinctions in folliculogenesis, induced-ovulator physiology, or species-specific mechanisms of cytoplasmic remodeling. Further research optimizing IVM conditions in camelids is warranted to determine whether such redistribution can be induced under controlled environments.

While distribution patterns (e.g., HoNP or HeNP) describe the macro-level spatial localization of mitochondria across the ooplasm, aggregation patterns (smooth or granular) reflect localized morphofunctional remodeling. Consequently, although granular aggregation frequently co-occurs with heterogeneous distributions such as HeNP, they remain distinct indicators of cytoplasmic reorganization. Regarding mitochondrial aggregation, the presence of clusters in 78% of oocytes suggests active structural and functional reorganization during maturation. In immature oocytes, smooth aggregations were associated with slightly higher fluorescence levels, potentially indicating a well-dispersed mitochondrial network capable of supporting early cytoplasmic functions [24]. In contrast, in mature oocytes, granular patterns correlated with increased fluorescence intensity, consistent with the heightened ATP requirements for spindle assembly and cytoskeletal reorganization [27]. Conversely, reduced mitochondrial activity in mature oocytes exhibiting smooth aggregation may indicate incomplete cytoplasmic maturation, which could negatively affect fertilization outcomes. In humans, mitochondrial clustering has been linked to improved fertilization rates and accelerated embryonic development [22], supporting the notion that granular aggregation in mature oocytes could represent a functional biomarker of competence.

Finally, the observed variability in fluorescence intensities across groups may reflect inherent biological differences among post-mortem–derived oocytes, potentially influenced by factors such as follicular atresia or prior hormonal exposure. Nonetheless, the distinct mitochondrial distribution and aggregation patterns observed in alpaca oocytes appear to hold promise as biomarkers for assessing cytoplasmic competence and developmental potential. These findings are not only relevant for understanding camelid oocyte biology but may also contribute to refining IVM protocols, improving embryo production, and developing genetic improvement and conservation strategies for South American camelids [28].

## 5. Conclusions

In conclusion, the characterization of mitochondrial activity, distribution, and aggregation patterns in immature and *in vitro* matured alpaca oocytes provides novel insights into the dynamics of cytoplasmic remodeling during maturation. The identification of specific patterns—such as the association of HeNP and granular aggregation with higher mitochondrial activity—highlights their potential as functional biomarkers of oocyte competence. Although further studies linking these mitochondrial parameters to fertilization and embryo development are required, our findings offer a valuable foundation for establishing evidence-based oocyte selection criteria and for optimizing IVM systems in alpacas. These advances could ultimately enhance reproductive biotechnologies and contribute to both genetic improvement and conservation strategies for South American camelids.

## Data Availability

Data supporting link: https://grupoeducad-my.sharepoint.com/:f:/g/personal/sevangelista_cientifica_edu_pe/IgAsE83wW8Q_R6-iYs7jeamfAbThU0oTLNwKEXyDsp25dOI?e=qU9GAu (accessed on 2 June 2026).

## Abbreviations

The following abbreviations are used in this manuscript:

COCs	Cumulus–oocyte complexes
HoNP	Homogeneous non-peripheral
HeP	Heterogeneous peripheral
HeNP	Heterogeneous non-peripheral
IVM	*In vitro* maturation
IVF	*In vitro* fertilization
